# Authority Relationship From a Societal Perspective: Social Representations of Obedience and Disobedience in Austrian Young Adults

**DOI:** 10.5964/ejop.v11i2.883

**Published:** 2015-05-29

**Authors:** Francesco Fattori, Simone Curly, Amrei C. Jörchel, Maura Pozzi, Dominik Mihalits, Sara Alfieri

**Affiliations:** a Department of Psychology, Università Cattolica del Sacro Cuore, Milan, Italy; b Department of Psychology, Sigmund Freud PrivatUniversität, Vienna, Austria; University of Neuchâtel, Neuchâtel, Switzerland

**Keywords:** social representations, obedience, disobedience, young adults, mixed-method approach

## Abstract

Obedience and disobedience have always been salient issues for both civil society and social psychologists. Since Milgram’s first studies on destructive obedience there has not been a bottom-up definition of what obedience and disobedience mean. The current study aimed at investigating the social representations young adults use to define and to co-construct knowledge about obedience and disobedience in Austria. One hundred fifty four (106 females, 68.8%) Austrian young adults (Mean age = 22.9; SD = 3.5) completed a mixed-method questionnaire comprising open-ended questions and free word associations. Overall obedience and disobedience are respectively defined as conformity and non-conformity to regulations, ranging from implicit social norms to explicit formal laws. Authority is multi-faceted and has a central role in orienting obedience and disobedience. Further fundamental determinants of the authority relationship and relevant application of the results are discussed in this paper.

## Introduction

The relationship with the authority is a fundamental element in every social context, since every social organization, whether it is a family or an institution, is based on an hierarchical structure to adequately function (Passini & Morselli, 2010c). The intrinsic hierarchical nature of authority relationship allowed the scholar to reduce complexity by referring to it in terms of obedience and disobedience (Blass, 2012; Bocchiaro & Zamperini, 2012; Milgram, 1965; Passini & Morselli, 2009). In fact obedience and disobedience can occur only within a hierarchical structure, as highlighted by Milgram (1974) in his differential analysis between obedience and conformism. Analyzing the state of the art within the authority relationship literature, Morselli and Passini (2011) moved further from classical studies on obedience and disobedience (Blass, 2012) defending the need for an analysis of obedience and disobedience as social objects and specifically that:

The authority relationship cannot be only considered an individual relational process, given that it has the function of regulating the life of the community. Without wishing to reject any of the individual level explanations, the argument here is that they should be integrated within a societal level approach, thereby highlighting the link between the two levels (p. 298).

Thus, the importance of considering the societal level in the authority relationship analysis, going beyond individual and not ecological inquiry, can contribute to a full comprehension of these complex phenomena (Morselli & Passini, 2011; Moscovici, 2011). According to these assumptions, several scholars (Elcheroth, Doise, & Reicher, 2011; Morselli & Passini, 2011; Staerklé, Clémence, & Spini, 2011) highlighted the relevance of studying authority relationship through the paradigmatic lenses of social representations theory (SRT) (Moscovici, 1961). In a concise definition, social representations are shared knowledge that people build together in order to act within the world (Abric & Tafani, 2009) and, according to these premises, this study aims to define and unveil the social representations of obedience and disobedience using a mixed-method research approach.

### Obedience and Disobedience as Socio-Psychological Research Objects

In 1936, a cross-cultural study investigating the phenomena of obedience and disobedience to authority was conducted (Fromm, Horkheimer, Mayer, & Marcuse, 1936). The aim of that study was to establish psychological structures that determined the dependency of individuals on societies’ rules. The focus was on the family because, according to the authors, the very first instance to come into contact or experience authority occurs within the family. Therefore the family has a crucial role in the development of one’s ability to assign and subordinate, reproducing the required social forms of living together and the necessary adaptation to the authorities, which are essential for the construction of social order. Furthermore, referring to a broader social context than the family, there is no doubt about the pivotal role of obedience for “the success of most human groups and organizations, which in turn is crucial to the biological success of our species” (Ent & Baumeister, 2014, p. 575). This societal level was neither considered nor analyzed by social psychologists who focused their studies rather on a specific behavior: destructive obedience (Burger, 2009; Milgram, 1963). Stanley Milgram conducted experimental research searching for situational conditions fostering destructive obedience. The findings suggested that people are more than willing to implement inhumane behavior if requested by an authority (in a precise situation, up to 95% of the participants gave potentially lethal electric shock to another human being). The Yale scholar admitted a “painful alternation in [one's] own thinking” (Milgram, 1965, p. 74) as he observed how many subjects experienced deeply stressful sensations, yet they complied. Since this extraordinary experiment, similar studies have been discouraged or even prevented by “sets of highly restrictive rules and regulations established by federal government” (Elms, 1995, p. 3). Despite heightened federal ethical regulations, Burger (2009) investigated the question whether people would still obey today with a milder version of the experiment and concluded that “average Americans react to this laboratory situation today as much the way they did 45 years ago” (Burger, 2009, p. 16). Milgram’s results were shocking but did not analyze disobedient behavior and explained only a small part of the authority relationship phenomena.

Only recently the interest has shifted to studying specific acts of disobedience to an authority, such as whistle-blowing (Bocchiaro, Zimbardo, & Van Lange, 2012; Fraschini, Parisi, & Rinoldi, 2011) and collective action (Thomas, Mavor, & McGarty, 2012). Examples of this specific disobedience can be seen in social actions such as *Occupy Hong Kong* or *the Arab Spring*. A question like “Why do people conduct gruesome tasks under the premise of obeying an authority?” has been the focus in studies such as Milgram’s (1965) destructive obedience study and has now shifted to “when do people disobey authorities in order to stand up for a better society?”

This unique focus on just one specific kind of authority relationship and, furthermore, only through experimental paradigm (Blass, 2012), left uncovered what Doise (1986) theorized as the ideological level of analysis. Only recently, a growing number of studies is addressing the inquiry of authority relationship according to a constructive methodology (Morselli & Passini, 2012; Pozzi, Fattori, Bocchiaro, & Alfieri, 2014). Morselli and Passini (2012) identified some fundamental components of the representations of obedience and disobedience as, respectively, *democracy*, *reciprocity*, *freedom*, *submission*, *passivity*, and *dictatorship* and *right*, *duty* and *transgression*. In a recent study (Pozzi et al., 2014) conducted according to the structuralist approach of social representations (Abric, 2003a), these two representations have been defined as composed by common themes such as *authority* and *norms*, but differentiating for issues such as the degree of activation of the subjects enacting obedience or disobedience.

Societies develop systems of beliefs, values, norms and representations to understand and interpret the social events. In order to understand the behavior of people disobeying authorities and not blindly following state rules and regulations, we must first understand how people represent the concepts of obedience and disobedience (Morselli & Passini, 2011). In fact, in addition to hierarchical contexts, people are included in communicative contexts within which transmit, shape and negotiate their knowledge, values and attitudes (Palmonari & Emiliani, 2009). It is relevant to unveil the representations of authority relationship because the way people mean it and represent it influence social change and democracy processes (Morselli & Passini, 2011). In fact, people, sharing meanings and scripts, give order to reality and use social norms to establish the adequacy of a behavior (Galli, 2006; Moscovici, 2011). In this sense, when representations became pervasive in a social group they can assume normative functions and define the institutional asset of that specific community (Morselli & Passini, 2011). Moreover many definitions of the authority relationship have been given throughout history and by different human sciences approaches (e.g. Fromm, 1981; Rattner, Yagil, & Sherman-Sega, 2003; Schlesinger, 1975; Thomassen, 2007) but these theoretical definitions were based on assumptions of logic and top-down empirical evidence.

According to these premises, what are the common meaning structures of the concepts of obedience and disobedience that guide and constrain actions? Which are the bottom-up definitions of obedience and disobedience? To fill this gap in the literature on authority relationship we chose social representations theory (SRT) (Moscovici, 1961) as the theoretical frame for studying authority relationship.

### Social Representations Theory (SRT)

The concept of social representation (SR), developed and advocated by Serge Moscovici (1984, 1988), plays a significant part in the field of social psychology as it focuses on how social (lay) knowledge is generated and influenced within the social environment and how it guides and constrains human behavior. While multiple variations of definitions exist also within Moscovici’s own work, it is correct to define social representations as a system of ideas, practices, images and values with their own cultural meaning; the knowledge is acquired directly through experiencing the behavior in the family, friends and school peers and it includes all processes of memory, perception, and information-gathering which work together to provide knowledge within a social context (Moscovici, 2001).

Within the field of SRT there have been multiple developments (Palmonari & Emiliani, 2009). Abric (1993) and his colleagues from the Aix-en-Provence school have theorized social representations as composed of a *content* and a *structure*. The content includes the information, beliefs and attitudes that a specific social group has referring to a social object. Structural elements instead show the organization of the content and can be distinguished between *nucleus* and *peripheral* elements, in terms of the centrality and stability of certain beliefs. This approach has come to be known as Central Nucleus Theory (CNT; Abric, 1994). Accordingly, SRs are organized around a stable central nucleus, which represents meaning and structure. As the central nucleus is the element most resistant to change, any modification of the central nucleus leads to a complete transformation of the representation. For two representations to be different, they must be organized around two different central *nuclei* (Galli, 2008). Furthermore, the organization of SRs is determined by the combination of two systems: the central system reflecting the social environment and knowledge as a collective basis; and the peripheral system which depends on characteristics of the individuals, and suggests more flexibility and the possibility of integrating and processing knowledge. As two representations could have the same content but different structure and then different meanings (Fasanelli, Galli, & Sommella, 2005), it is therefore fundamental to study both the content and the structure to enrich the comprehension of a social representation.

To summarize, the aims of the present paper are: (a) to fill the gap in social psychology literature, giving a completely bottom-up definition of these two social objects; (b) to investigate the components defining obedience and disobedience to conduct a differential analysis and to highlight common themes and differences; and (c) to have scientific clues to further analyze, understand and interpret specific political psychological phenomena, such as current social protests (Elcheroth et al., 2011).

## Method

### Participants

One hundred fifty four participants, mostly psychology students at the Sigmund Freud PrivatUniversität (SFU - Vienna), were recruited and entered in the study on a voluntarily basis, signing participation consent. Their mean age was 22.9 (*SD* = 3.5) and 106 participants were female (68%). Our sample was composed of: 97 university students (63%), 28 student-workers (18.2%), 9 workers (5.8%), 15 unemployed or other (9.7%). Seventy-one participants (53 females, 74.6%) completed the task referring to obedience while 83 (53 females, 63.85%) participants answered the questionnaire related to disobedience.

### Measures and Research Design

Participants were administered a self-report questionnaire composed of:

1. An open-ended question aiming to investigate the content of the representation. Participants were asked to report how they define obedience or disobedience (“In your opinion, what is obedience?” and “In your opinion, what is disobedience?”).
2. An exercise of free association aimed at uncovering the structure of the representation. Participants were asked to associate five nouns and five adjectives to the inductor term (obedience or disobedience according to casual assignation to sub-samples). Then they were asked to explain their choices in order to disambiguate the meaning of the selected terms. After this exercise, participants were asked to rank by importance, from 1 to 5, the terms freely associated. This procedure was run in accordance with the Hierarchized Evocation Technique (Vergès, 1992) in order to find nuclear and peripheral elements.

With the open-ended question, we obtained qualitative data that were analyzed using thematic analysis (Braun & Clarke, 2006). The data set collected through free association (see Point 2) was analyzed by running Evoc2000 software (Vergès, 1992), resulting in the structural organization of the content.

The thematic analysis method (Braun & Clarke, 2006) allows the researcher to extract the main components and themes forming the content of the representations. A theme represents patterned responses from the data that are relevant to the research question. Thematic analysis include the following steps: familiarization of the data set, generating initial codes, identifying themes amongst codes, and defining and deducting themes into the summarized final narrative reports (Braun & Clarke, 2006).

The structure of the representation was generated by running the free association answers through the Evoc2000 software. This software runs in accordance with TCN (Abric, 2003a; Vergès, 1992) by crossing two criteria of prototypicality: the *frequency* of appearance and the *rank* of its importance, that is, the average position in which a word is classified. Evoc2000 software generates a four-quadrant matrix taking into account the criteria of word frequencies and order of evocation (Abric, 2003a). For example, the central nucleus (top left quadrant) is generated by words with the highest frequencies and ranked as most salient.

This procedure is in accordance to the assumption that “only the intersection of these two [qualitative and quantitative] criteria allows for the identification of the statute of constitutive elements of the social representation being studied” (Fasanelli et al., 2005, p. 113). The results come from the agreement of three independent researchers who completed the analysis of the categories obedience and disobedience. After a first phase of individual work on raw data, all three researchers compared their findings. The final analysis resulted from the comparison and the negotiated integration of the three analyses.

## Results

### Obedience

#### Thematic Analysis

The analyses of the responses to the open-ended question yielded six labels (see Table 1). Obedience was defined as conformity with regulations, acceptance of laws without any questioning, and the ability to contemplate one’s conformity with regulation was stressed among the answers. Especially in the upbringing of children, finding the middle ground seems crucial. There was mention of the fear of punishment when standing against or refusing to obey regulations. Obedience was also seen as a sign of respect towards other people, whether they are children, parents, or older people. Regulations were divided into rigid laws, social norms and conventions, within the family or school. Obedience meant losing the possibility or quality of being oneself, being different from other people, while still recognizing the importance of obedience for the functioning of society. It also implies solidarity and protection of other members of the society. Distinction between positive and negative aspects of obedience was contemplated.

**Table 1:** Thematic Analysis – Obedience

Themes	Themes’ definition
Conformity	Obedience is perceived as conformity with regulations, “to carry out commands” (Participant 1). It is the willingness to follow and accept regulations and laws, for some participants it means to “take orders and carry them out to 100%” (Participant 22). One must adjust to the society, but it is also seen as a ranking of importance.
With/out Reflection	In this category thoughts on obedience are summarized with/without reflection. It means to follow a person, regulation, or system without thinking about one’s reasons or behavior. For some participants it is a question of finding middle ground, to “Follow without thinking about meaningfulness“ (Participant 3). Especially, in the upbringing of children it is important to find the right way of dealing with obedience “the right measure is the deciding factor” (Participant 37).
Fear of Punishment	Obedience is associated with a fear of punishment. This fear is a reason why people obey the law and submit to an authority. But it also is seen as a sign of respect, some set obedience as equal to respect “Reason for obedient behavior is fear” (Participant 4).
Types of Authority	Participants perceive obedience to different types of authority. Some see it as an acceptance of regulations and obligations; others identify authority in the form of social norms and conventions, to “observe the regulations and obligations” (Participant 2). There is a blind and abstract obedience, and the word is connected to educating children, the process of bringing up “students who follow at school, children observe rules of parents” (Participant 23).
No-Individuality	For many participants, obedience means no individuality because “thoughts and personal opinions do not matter” (Participant 1). One might also obey out of self-compulsion or out of responsibility to protect other members of society.
Positive/negative Connotation	Another opinion on the subject is the distinction between positive and negative connotation of obedience, as well as healthy and unhealthy obedience. In some cases obedience can be dangerous “there is healthy and unhealthy obedience” (Participant 78).

*Note.* The labels presented are listed in rank according to their frequency beginning with the label that scored the most answers.

#### Structure Analysis

In Table 2 the free word associations to the term obedience are organized according to Evoc2000 output. While analyzing this table please refer to the following explanation: in the nucleus, upper left quadrant, characterized by a high frequency of appearance and by a high average rank of appearance, we have those terms constituting the core meaning; which gives unity and stability to the representation. In the first periphery, upper right quadrant, characterized by a high frequency of appearance and by a low average rank of appearance, we have those terms indicating behavioral tendencies (Abric & Tafani, 2009). In the element of contrast quadrant, in the lower left angle, low frequency and high average rank, we have those terms representing a minority group’s beliefs. In the last quadrant, lower right angle, low frequency and low average rank, we can observe those elements fading in or out from the representation.

**Table 2:** Structure Analysis – Obedience

Nucleus			First periphery		
Word	Frequency	Rank	Word	Frequency	Rank
*Noun (Frequency ≥ 14; Rank ≤ 2.6)*			*Noun (Frequency ≥ 14; Rank ≥ 2.6)*		
authority	19	2.3	upbringing	32	3.1
regulations	17	2.3			
power	16	2.2			
*Adjective (Frequency ≥ 7; Rank < 2.6)*			*Adjective (Frequency ≥ 7; Rank ≥ 2.6)*		
blind	9	2.0	obedient	40	2.8
nice	9	2.6	anxious	7	3.3
submissive	7	2.1			
important	7	2.6			
Elements of contrast			Second periphery		
Word	Frequency	Rank	Word	Frequency	Rank
*Noun (5 ≤ Frequency < 13; Rank < 2.6)*			*Noun (5 ≤ Frequency ≤ 13; Rank ≥ 2.6)*		
command	10	2.5	suppression	13	3.1
laws	10	2.5	consequences	11	2.8
compulsion	7	2.4	military	10	2.9
discipline	6	2.3	punishment	9	3.6
respect	6	2.3	subordination	6	3.2
will	5	2.4	violence	6	3.5
			implementation	5	3.2
*Adjective (4 ≤ Frequency < 6; Rank < 2.6)*			*Adjective (4 ≤ Frequency ≤ 6; Rank ≥ 2.6)*		
authoritarian	5	2.4	subordinate	6	3.2
restrained	5	2.4	adjusted	5	2.6
structured	4	1.5	restrictive	5	2.6
			weak-willed	4	3.2
			punishing	4	3.2
			strict	4	3.5
			positioning	4	4.0
			disciplined	4	4.7

### Disobedience

#### Thematic Analysis

The evaluation of the data demonstrates the complexity of this social object. The responses of the participants were reduced into nine labels as depicted in Table 3. The majority perceived disobedience as non-conformity with existing regulations or authority regardless of whether that is a person or a system. According to their answers, authority can take on several forms, varying from familial surroundings, the political arena, institutional policies, social expectations, but also self-inflected rules. In particular, many participants associated disobedience as a phenomenon occurring within the familial context. Another factor often mentioned in the answers refers to the ability to determine and control one’s actions. It implicates either a conscious and unconscious judgment or decision to oppose authority. Individuality can also imply seeking attention or dealing with frustration. A superior-inferior relationship in which power is established is already a premise to accept and perceive authority. Positive and negative aspects of disobedience have been collaborated upon, stating that in some situations or contexts obeying is just as important as disobeying. Another way to describe disobedience is the reduction to that of “opposite of obedience” because only in connection with obedience, disobedience is possible.

**Table 3:** Thematic Analysis – Disobedience

Themes	Themes’ definition
Non-conformity	The majority of participants sees disobedience as non-conformity with regulations, “to oppose regulations and laws, to disregard regulations and standards” (Participant 2), regardless whether these regulations make sense or not. In the case of regulations being absurd, non-conformity with laws is legally justified and “it lies in the hands of the acting person and not in the hands of the superior” (Participant 45).
Types of authority	Disobedience is perceived with different types of authority, such as social, political, familial or institutional policies. More semantic difference is made between family and public “disobedience only at home, means violating of regulations” (Participant 3). Some perceived authority in the form of conventions “not to hold on to social norms” (Participant 5); others even refer to regulation as “advice and opinion of others” (Participant 75).
Self-Mastery (determination)	Opinions belong in this category of self-mastery that reflect disobedience as an act of self-determination, when one decides his/her destiny without accepting the commands from others “doing his own thing” (Participant 6). It also can be seen as an attempt to be different, special, or not following the mainstream, “to be unique” (Participant 5). It can result in one`s frustration or be used to test boundaries.
Conscious/unconscious	The different approach towards disobedience shows the answers of those participants who mentioned conscious-unconscious reactions to disobedience “A conscious or unconscious protest against demands made by a superior person or a system (Participant 35).
Negative/positive connotation	There is a distinction concerning disobedience between negative and positive connotation. Some participants connect it to negativity because it reminds them of negative authority, and others to positivity because they refer to rebellion. One participant has more objectivity when he expresses his thoughts on the subject as “Whether an action is to be evaluated as positive or negative depends on the case” (Participant 10).
Hierarchy	Disobedience has something to do with hierarchy as well. There has to be a superior-inferior relationship in order to act as an authority. Without such a presumption there is no disobeying, “not following superiors’ instructions” (Participant 34).
Force and punishment	Disobedience is associated with force and punishment. For some participants suppressing disobedience violently makes it possible to gain power over others “The alleged authority becomes real when disobedience is violently suppressed” (Participant 22). To stand up against authority can and will be punished, many associate disobedience in the family context, as in teaching or educating children to obey. Disobedience can bring sanctions” (Participant 58). It is also seen as a lack of respect for authority, regardless of whether in public or in the family.
Future orientation	The category future orientation reflects on disobedience as an important part of an upbringing. It can have negative consequences for the future if children do not learn it in childhood “belongs to growing-up, to learning for the future (Participant 25).
Opposite of obedience	Some participants expressed their thoughts on disobedience as the opposite of obedience, claiming that only “in combination with obedience is a definition possible” Participant 62).

#### Structure Analysis

In Table 4 the free word associations to the term disobedience are organized according to Evoc2000. Please refer to the previous section "Obedience", subsection "Structure Analysis" for the explanation of the quadrants’ meanings.

**Table 4:** Structure Analysis – Disobedience

Nucleus			First periphery		
Word	Frequency	Rank	Word	Frequency	Rank
*Noun (Frequency ≥ 13; Rank ≤ 2.6)*			*Noun (Frequency ≥ 13; Rank ≥ 2.6)*		
regulations	21	2.1	upbringing	37	2.8
rebellion	17	2.6	resistance	37	2.8
					
			punishment	23	3.4
			individuality	19	2.8
*Adjective (Frequency ≥ 12; Rank < 2.6)*			*Adjective (Frequency ≥ 12; Rank ≥ 2.6)*		
brave	17	2.1	against	61	2.9
reflective	12	2.4	negative	31	3.2
			regulated	27	3.0
			individual	19	2.7
			rebellious	18	2.9
			disrespect	14	3.2
Elements of contrast			Second periphery		
Word	Frequency	Rank	Word	Frequency	Rank
*Noun (6 ≤ Frequency < 12; Rank < 2.6)*			*Noun (6 ≤ Frequency ≤ 12; Rank ≥ 2.6)*		
spite	10	2.3	stubbornness	12	3.7
regulation breach	8	2.0	freedom	8	2.6
			authority	8	2.7
			politics	7	3.3
			courage	6	3.2
			obedience	6	4.0
*Adjective (6 ≤ Frequency < 11; Rank < 2.6)*			*Adjective (6 ≤ Frequency < 11; Rank ≥ 2.6)*		
self-confident	9	2.4	young	10	3.2
free	6	2.3	selfish	7	3.3

## Discussion

### Obedience

Results indicate a good correspondence between the thematic and structural analysis. The concept of obedience evoked the notion of conformity with regulations, acceptance of laws without question, while recognizing the importance of obedience for social order and the functioning of society. This definition is reinforced with terms from the nucleus: *regulation* and *submissive.* The former refers to the need of norms to be followed while the latter refers to the hierarchical position of the obedient person. One of the main theoretical components of obedience, which differs from conformism, is hierarchy (Passini & Morselli, 2010b): for the concept of obedience, it is necessary to have a person with a higher status, an *authority* (nucleus term), otherwise we are dealing with conformism, regulating social influence between peers.

In our data authority is conceived as a two-fold object: it is represented as a physical authority giving orders or as a social entity composed of a set of social norms, regulations and conventions. Regarding physical authority, parents are the main actors objectifying it. Family^i^ is the first social context requiring obedience from its members and it is the first place in which obedience is socially learned by children as future citizens (Darling, Cumsille, & Loreto, 2007; Fromm et al., 1936). The family, therefore, has a crucial role in developing the ability to comply and subordinate, reproducing the required social forms of living together and the necessary adaptation to the authorities which are essential for the construction of public order (Xiao, 1999). Within this perspective, it is pivotal for parents not to raise children as obedient machines who turn out to be future “sleeping” citizens, blindly accepting the authorities’ requests (Fromm, 1981) that can lead to negative social consequences.

Within our data the notion of obedience further evoked different types of regulations, ranging from the most rigid to more lenient ones: rigid laws, social norms and conventions, and rules within the family or school. Interestingly every category of regulations belongs to a specific authority, whether it is an informal order from a parent or a formal law written in the Constitution.

Furthermore, obedience evoked the issue of individuality and reflection. Within the literature several theories link obedience to blind submission, explaining this relationship as resulting from lacking individual reflection in response to authority’s requests (Bandura, 1990; Bocchiaro et al., 2012; Milgram, 1974; Pozzi et al., 2014; Zimbardo, 2004).

Recently, the blind nature of obedient behavior has been discussed and Milgram’s experiments re-analyzed, looking for further explanatory variables. Results indicate, for example, that the participant’s willingness to contribute to the further progression of science was a fundamental component of their destructive behavior (Haslam, Reicher, & Birney, 2014; Reicher, Haslam, & Miller, 2014).

Another important theme outlined in our results is that fear of *punishment* encourages obedience (Kelman & Hamilton, 1989). Ideally authority should govern without the use of punishment; exercising coercion usually is a sign of losing power over citizens and punishment becomes necessary in order to preserve the status quo. “Social systems cannot be undermined each time the authority lacks legitimacy and its influence is not strong enough to guarantee a proper level of obedience. Thus, disobedience to authority is limited and discouraged by establishing sanctions and punishments” (Morselli & Passini, 2011, p. 294).

In our data set obedience evoked a consequence-based evaluation: it is not positive or negative *per se* but depends on the fairness of its actions and consequences. This result is coherent with recent definitions of obedience (Passini & Morselli, 2009): in this respect obedience can be defined as constructive when it preserves social order and destructive when its actions have negative consequences for people, groups or communities.

### Disobedience

The majority of the participants conceived disobedience as non-conformity with existing regulations or authority, regardless of whether these are persons or a social system. Rebellion to unjust regulations, in terms of making a stand and not follow rules or formal laws despite possible consequences, requires courage and reflective skills. Reflection is a component characterizing disobedience (Pozzi et al., 2014) and it is connected to disobedience to the extent to which a person is able to process an authority’s request and answer accordingly to universal ethical standards (Bocchiaro & Zimbardo, 2010).

In our data set the associations to disobedience were described as unconscious^ii^ reactions to regulations or authority requests. This dichotomy in the reflective nature of disobedience could be due to “a difference in their interpretation of the situation as one requiring a new immediate action on their part, of perceiving danger, or threat, or immorality that others may misidentify as less urgent” (Bocchiaro & Zimbardo, 2010, p. 167).

Within our results disobedience was related to authority figures that can take several forms, varying from familiar surroundings, the political arena, institutional policies, social expectations, to self-inflected rules. Authority and hierarchy are binding requirements for disobedience, both on a personal and a community level (van Zomeren, Spears, Fischer, & Leach, 2004). When an authority and its requests lack legitimacy, disobedience is likely to occur and punishment is the easiest strategy an authority can use to maintain the status quo and its power (Passini & Morselli, 2010a). Sanctions and punishment, both on a personal and a social level, such as stigmatization, either foster or inhibit a person’s disobedient behavior (Zimbardo, 2007). Yet, within the civil disobedience theoretical framework, disobedient behavior differs from deviant behavior in respect to dealing with punishment: persons acting in civil disobedience accepts the consequences of their actions, using punishment as an amplifier of their political message (Arendt, 1985).

On a personal level disobedience is strictly connected to self-determination in our data set; every person has the right and the duty to decide destiny. This issue has been discussed within the literature on the individual level, recalling the highest level of Maslow’s pyramid^iii^ (1943), but also at the socio-political level, for example community self-determination (Wellman, 1995). While the former refers to persons’ needs of reaching their full potential, whatever it may be, Community self-determination refers to the need of people belonging to the same ethnic group to re-draw national boundaries, to fulfill secession from its state, and to consequently annex to another state. These two processes and their respective aims often require disobedience: in our first case towards parents, social groups, or norms and in the case of community self-determination to national laws.

In the latter case, rebellion or resistance may be enacted. While perceived as disobedience against rules and laws, also in case of injustice, they are acts of violation of a legal directive that can be justified when fundamental rights within a democracy are at stake (Moraro, 2014).

In correspondence with other studies on social representations of authority relationships in different cultural contexts (Morselli & Passini, 2012; Pozzi et al., 2014), our results also show that evaluations of disobedience are consequence-based. While it has a negative connotation when it leads to disrespecting other people for one’s own benefit, it is considered positive when referring to rebellion for positive social changes (Passini & Morselli, 2009). In this respect disobedience is seen as positive whether related to important ethical issues, such as nuclear weapons (Buttle, 1985), or aimed at increasing fundamental rights and well-being to disadvantaged social groups (Thomas et al., 2012; van Zomeren, Leach, & Spears, 2012). Accordingly, rebellion is a nuclear element of disobedience and defines its necessary behavioral component (Zimbardo, 2007).

Further confirmed in this study, disobedience as social representation has an intrinsic bond with obedience (Morselli & Passini, 2012). This relationship can be theorized referring to Marková’s *themata* (Marková, 2003). The Czech scholar theorized common sense as composed of oppositional taxonomies (e.g. simplicity/complexity or analysis/synthesis) that can arise and become social representations under certain socio-historical conditions (Marková, 2000). The current historical period is characterized by evident action of civil and pro-social disobedience that could have thematized the obedience-disobedience antinomy: “Themata are such oppositional categories which, in the course of history, become problematized; for one reason or another they become the focus of attention, and a source of tension and conflict” (ibidem, p. 446).

Coherently, actual disobedient and protest actions can be interpreted referring to the social representations of obedience and disobedience described in this paper. Authority relationship is here considered as a dynamic relation between the individual and the authority, between the will of self-mastery of the individuals and the limits imposed by the authority. As recalled by Passini and Morselli (2010c), a functional democracy is possible in the balance between obedience and disobedience. People protesting in these years for different causes (Economic crisis, authoritarian governments, lack of free of speech, etc.) aim to decide for their future, consider authorities as illegitimate and strive for their self-mastery using disobedience as an instrument of active citizenship to change the unjust status quo (Passini & Morselli, 2011).

## A Definition of Obedience and Disobedience

The structure and thematic analysis complement each other for both obedience and disobedience. The two SRs have several common themes: (a) (Non) Conformity with regulations; (b) Multi-form regulations, ranging from formal laws to implicit social norms; (c) Authority is a multi-faceted object. It can be a physical person, an institution or a social entity; (d) Evaluation is context-based. Obedience and disobedience cannot be evaluated *per se* but according to the social consequences of their actions; and (e) Punishment is a strategy both to maintain obedience and to contrast disobedience.

The main difference resulted in the degree of reflection attributed to obedience and disobedience. Whereas obedience can be blind and enacted without reflecting on authority’s requests, disobedience always evokes a certain degree of reflection. This issue is connected with the individuality theme. Obedience evoked a lack of individuality, recalling Milgram’s (1974) agentic state, while disobedience is defined as a pathway to reach self-mastery (Maslow, 1943). According to our results we can give a completely bottom-up definition of obedience and disobedience. Obedience is conformity with a multi-form of regulations, ranging from implicit social norms to written formal laws, given by different authorities’ actors: people, groups, institutions, and society. Obedience is evaluated according to its consequences.

Disobedience on the other hand is a lack of conformity to multi-form regulations created by multi-faceted authorities, often characterized by a high degree of reflection and enacted at personal and community level.

## Conclusions

This study analyzed the authority relationship, namely obedience and disobedience, according to a social representation structural approach (Abric, 1994) to give bottom-up definitions of these two social objects, comparing their similarities and differences.

In our opinion, as authority relationship is a social object that people deal with from childhood onwards and as it is a social phenomenon currently characterizing the socio-political world, it had to be investigated according to a constructivist approach (Gelo, 2012), such as social representation theory. Furthermore, this study followed the recent reflections assuming the strict bond between social knowledge and political behavior (Morselli & Passini, 2011; Staerklé, 2009). The fundamental premise is the citizens’ inclusion in a specific political culture, permeated by a system of beliefs, ideologies and social values that are shared and that contribute to shape social behavior.

Possible limitations of this study could be the use of a single technique, including both open ended questions and free associations, to unveil the social representations of the authority relationship. The open-ended question should ideally replace, in a short and parsimonious form, the semi-structured interview that is often adopted to uncover the representation content (Fasanelli et al., 2005). Despite numerous existing techniques to retrieve social representations according to the structural approach (Abric, 2003b), this instrument allowed us to reach a broad sample and is an efficient alternative for conducting a rigorous, reliable and economical study. Another limitations regards the sampling procedure because, despite of this selection was run according to Moscovici’s indication of choosing a specific social group within the which inquiring a social object (Galli, 2006), the sample was mainly composed by university students of a psychology course. Being acquainted with psychological knowledge and theories (i.e. Milgram experiment or Freud theories) could have influenced the results. In order to overcome this limit, it is suggested to conduct further research on different samples, in order to verify the redundancy of the results.

The claim of saturating the analysis and the comprehension of a concept so rich in content surely cannot be satisfied by a single study. In this respect future research should focus on integrated approaches at a methodological level (Clark & Creswell, 2011) and the societal level has to be considered as one of the fundamental fields of inquiry to understand the current real social world more accurately.
